# ELA-Net: An Efficient Lightweight Attention Network for Skin Lesion Segmentation

**DOI:** 10.3390/s24134302

**Published:** 2024-07-02

**Authors:** Tianyu Nie, Yishi Zhao, Shihong Yao

**Affiliations:** 1 School of Geography and Information Engineering, China University of Geosciences, Wuhan 430074, China; niety@cug.edu.cn; 2 School of Computer Science, China University of Geosciences, Wuhan 430074, China; zhaoyishi@cug.edu.cn; 3 Engineering Research Center of Natural Resource Information Management and Digital Twin Engineering Software, Ministry of Education, Wuhan 430074, China

**Keywords:** skin lesion segmentation, lightweight network, attention mechanism, deep learning, medical image processing

## Abstract

In clinical conditions limited by equipment, attaining lightweight skin lesion segmentation is pivotal as it facilitates the integration of the model into diverse medical devices, thereby enhancing operational efficiency. However, the lightweight design of the model may face accuracy degradation, especially when dealing with complex images such as skin lesion images with irregular regions, blurred boundaries, and oversized boundaries. To address these challenges, we propose an efficient lightweight attention network (ELANet) for the skin lesion segmentation task. In ELANet, two different attention mechanisms of the bilateral residual module (BRM) can achieve complementary information, which enhances the sensitivity to features in spatial and channel dimensions, respectively, and then multiple BRMs are stacked for efficient feature extraction of the input information. In addition, the network acquires global information and improves segmentation accuracy by putting feature maps of different scales through multi-scale attention fusion (MAF) operations. Finally, we evaluate the performance of ELANet on three publicly available datasets, ISIC2016, ISIC2017, and ISIC2018, and the experimental results show that our algorithm can achieve 89.87%, 81.85%, and 82.87% of the mIoU on the three datasets with a parametric of 0.459 M, which is an excellent balance between accuracy and lightness and is superior to many existing segmentation methods.

## 1. Introduction

Skin cancer is a serious malignant tumor and ranks among the most prevalent forms of cancer worldwide [1]. The primary types of skin cancer include basal cell carcinoma, squamous cell carcinoma, and melanoma. Melanoma, while accounting for less than 5% of all skin cancers, has the highest malignancy and mortality rates [2]. An important consensus has appeared that early detection and treatment during screening can significantly increase the survival rate for melanoma patients, with rates reaching up to 90% [3]. Dermoscopy, a non-invasive technique, plays a pivotal role in examining and assessing skin lesions [4]. It aids in the diagnosis and treatment of skin disorders by magnifying images of the skin’s surface and providing physicians with a detailed view of its fine structure. Research has demonstrated that dermoscopy can substantially enhance diagnostic accuracy [5,6]. However, as skin lesions become increasingly intricate, manual examination of dermoscopic images becomes a laborious and time-intensive endeavor. It heavily relies on the operator’s expertise and dexterity, and any lapses in the process can lead to misdiagnoses or omissions. Consequently, computer-aided diagnosis (CAD) systems have been developed to elevate diagnostic accuracy and efficiency.

CAD systems have been widely used in the medical field and have become important tools in many hospitals for detecting skin diseases. Within the workflow of CAD systems, precise segmentation of lesion regions in skin images stands as a critical element. Early segmentation techniques primarily include thresholding [7], edge detection [8], and clustering [9]. Nonetheless, these methods are highly sensitive to initial thresholds and noise, resulting in the instability of segmentation outcomes. Furthermore, their computational efficiency is relatively subpar.

With the rapid development of deep learning, current segmentation methods based on Convolutional Neural Networks (CNNs) facilitate the progress of biomedical segmentation. Most of the methods attempt to construct models with high complexity to obtain multi-scale information, thereby achieving excellent segmentation accuracy. For instance, Deeplabv3+ [10] introduces atrous convolution with different dilation rates to expand the sensory field. PSPNet [11] proposes a pyramid pooling module to capture contextual information. EncNet [12] adopts a combination of atrous convolution and multi-scale strategy to achieve excellent performance on multiple datasets. In addition, U-Net [13]-based segmentation methods, including Attention U-Net [14], U-Net++ [15], V-Net [16], and U-Net v2 [17], are developed to enhance feature propagation.

In addition, the detection of skin lesions by whole-body system images is another research idea, which is characterized by the possibility of obtaining global-scale prediction data and is suitable for large-scale screening and disease surveillance, but since whole-body system images involve skin images from different body parts and under different lighting conditions, this diversity may pose a challenge for lesion segmentation. In current research practice, it is common practice to apply machine learning methods to whole-body system images, and CNNs are widely used to process whole-body system image data [18,19,20]. However, researchers have found that this method can be less effective in predicting smaller lesion regions than larger lesion regions [21], so methods that utilize whole-body image detection lack fine-grained prediction ability, and in practical application, appropriate methods should be selected according to specific needs and research purposes as a way to enhance work efficiency.

**Figure 1:**
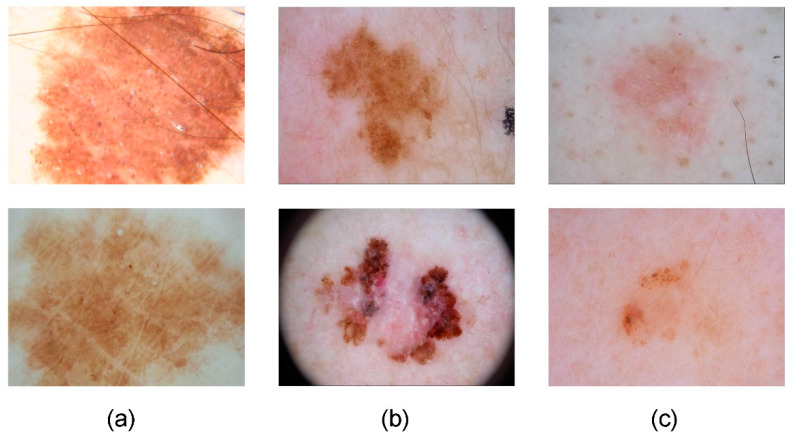
Some images of the public skin lesion dataset, ISIC2018. (**a**) Image of a lesion with oversized boundaries, (**b**) Image of a lesion with irregular lesion areas, (**c**) Image of a lesion with blurred boundaries.

The current hospitals may face limitations in terms of economic budget and hardware resources, potentially constraining their ability and willingness to deploy the high-complexity models that require substantial computational power. Therefore, it is necessary to design a lightweight segmentation model [22,23] so that it can achieve skin disease segmentation in resource-limited situations. However, due to the relatively simple structure and limited design of general lightweight models, when dealing with complex images of skin diseases (with large lesion boundaries (Figure 1a), irregular lesion areas (Figure 1b), low image contrast, making it difficult to define the lesion area and surrounding tissue boundaries (Figure 1c) and other issues), it is difficult to capture subtle edge features, thereby impacting the accuracy of segmentation tasks and resulting in potential issues like misjudgments and missed detections. Consequently, there is a critical need to design a segmentation model that is both lightweight and high-accuracy, aiming to offer more dependable support for the analysis and diagnosis of medical images in clinical settings.

In this paper, we present an efficient lightweight attention network (ELANet) for skin lesion segmentation. The core component of this network is the bilateral residual module (BRM), which forms the backbone architecture of the network by connecting a number of BRMs in series through three downsampling modules, and this structure enables efficient feature extraction from the input image with very low computational complexity. Particularly, the semantic and spatial information of the input stream is particularly important during network processing. For this reason, two attention mechanisms, channel and spatial, are embedded in the two branches of the BRM to fully capture the feature information of the image so as to enhance the segmentation accuracy of the model. In addition, we unite feature maps of different scales and adopt the strategy of multiscale fusion to achieve the multiplexing of feature maps, which can enrich the receptive domain of the network and expand the receptive field. Finally, at the end of the network, feature maps with sufficient information are processed using the pyramid pooling module (PPM), which helps the network learn more comprehensive and detailed features and achieves improved segmentation accuracy with low computational overhead.

In summary, the innovation of our work lies in the design of a novel module (BRM) for extracting skin lesion image features and combining it with a multi-scale strategy to construct a segmentation model, ELANet, which can help physicians diagnose skin lesions more accurately and improve the efficiency and accuracy of treatment.

The main contributions of this paper can be summarized as follows:

1. We propose a lightweight segmentation model, ELANet, which achieves efficient skin lesion segmentation with an extremely low parameter, facilitating its deployment in resource-constrained clinical devices.
2. We design the BRM for seamless integration into the model. Its internal atrous convolution and attention mechanisms comprehensively capture feature information, thereby enhancing the model’s segmentation accuracy.
3. We construct a multi-scale attention fusion (MAF) module that combines output streams of various scales, effectively solving the information loss caused by down sampling operations and obtaining global context information.
4. We conduct extensive experiments on three public datasets, ISIC2016, ISIC2017, and ISIC2018, achieving state-of-the-art performance and attaining an excellent balance between accuracy and lightness.

## 2. Related Work

In this section, we provide an overview of the challenges faced in the field of skin lesion segmentation as well as related research advances. The main focus is on three key aspects: methods for skin lesion segmentation, lightweight networks, and attention mechanisms. Related work in these three areas is described below.

### 2.1. Skin Lesion Segmentation

Currently, CNN-based models have been widely used in the field of dermatological segmentation. Inspired by the fully convolutional network (FCN) [24], Yu et al. [25] first devised the fully convolutional residual network (FCRN), enhancing segmentation performance through the integration of multi-scale contextual information. Bi et al. [26] then proposed an automatic skin damage segmentation method based on FCN architecture and introduced deep class-specific learning to overcome the feature ambiguity. Subsequently, Esfahani et al. [27] developed DPFCN, featuring a novel dense pooling layer that achieves high-accuracy segmentation of skin images. Tang et al. [28] proposed a method for skin lesion segmentation (SLS) utilizing the separable-UNet architecture with stochastic weighted averaging, aiming to improve pixel-level discriminative representation. Arora et al. [29] developed a custom U-Net architecture with Attention Gates-Based design, incorporating atrous convolution to expand the receptive field. Wu et al. [30] introduced a network based on a dual encoder architecture, incorporating the adaptive dual attention module (ADAM), for automated skin lesion segmentation from dermatoscopic images. Abhishek et al. [31] designed a deep semantic segmentation framework for dermatoscopic images by augmenting the RGB dermatoscopic image with additional color bands and intrinsic, greyscale, and shadow-attenuated images. 

However, the above approaches focus on segmentation accuracy while ignoring the variations in parameter quantity, resulting in large parameters in the model, which is detrimental in clinical conditions where lightweight deployment is required. Hence, our algorithms focus on lightweight designs that reduce the number of model parameters to enable deployment in low-memory practical applications.

### 2.2. Lightweight Networks

The utilization of complex model architectures tends to put a considerable load on computing devices. To tackle this challenge, researchers have initiated investigations into the design and optimization of lightweight networks. Enet [32], introduced by Adam et al., emerged as one of the pioneering networks tailored for lightweight real-time segmentation. Its compact structure is well-suited for deployment on embedded devices. Building upon Enet, Romera et al. [33] proposed ErfNet, which incorporates residual concatenation and factorization convolution, yielding more precise outputs. Recently, Jeya et al. [34] proposed a lightweight network called UNext, which combines the MLP and UNet structures to reduce the number of model parameters while ensuring accuracy. In addition, MALUNet [35], designed by Ruan et al., is also based on the U-shaped structure and introduces various attention mechanisms to reduce the model size, achieving excellent performance in the skin lesion segmentation task. They also proposed another lightweight model, EGEUNet [36], which uses the grouping idea to capture information from different perspectives, and the model solves the skin lesion segmentation task with only 50 KB parameters.

The common approach to lightweight modeling involves reducing the network’s depth or the number of neurons per layer. However, such simplifications can hinder the model’s capacity to fully capture essential information, thereby affecting the model’s predictive ability. To address this issue, we introduce an attention mechanism that enhances the model’s ability to perceive features without increasing additional computational overhead, thereby improving overall performance. It meets the dual requirements of accuracy and lightness in practical application environments.

### 2.3. Attention Mechanisms

Attention mechanisms have proven to be useful tools for neural networks, facilitating the learning of essential information and enhancing a model’s predictive capabilities. Wang et al. [37] introduced the residual attention network, which analyzes image features by stacking attention blocks. Jaderberg et al. [38] devised the spatial converter, enabling the network to operate on feature maps in the spatial domain. On the other hand, Hu et al. [39] focused on channel relations and proposed Squeeze and Excitation (SE) blocks to re-model the interdependence between channels. However, both spatial and channel attention omit some feature information; therefore, Sanghyun et al. [40] combined these two dimensions and proposed CBAM, which sequentially infers the attention graph along two independent dimensions (channel and spatial) to achieve a more comprehensive resource allocation. In addition, Kaul et al. [41] also designed FocusNet through a hybrid attention mechanism, which achieved good results in the fields of skin cancer and lung lesion segmentation. 

Across various levels of neural networks, there are differences in the spatial and semantic information contained in feature maps, which provide the spatial position and category information of the target, respectively. However, embedding only one type of attention mechanism will ignore another type of feature information, which cannot meet global needs. Therefore, in this paper, we design a hybrid attention mechanism that concurrently incorporates both spatial and channel attention mechanisms. This innovative approach enables the model to pinpoint the target’s position and capture its category features simultaneously, thereby enhancing the model’s performance in skin lesion segmentation tasks.

## 3. Methods

In this section, we will first describe the overall architecture of ELANet and its three core components, including the downsampling module (DSM), the pyramid pooling module (PPM), and the feature fusion module (FFM). Then the multi-scale attention fusion operations used by the network are described, after which the structure of the core module BRM will be given.

### 3.1. ELANet Architecture

**Figure 2:**
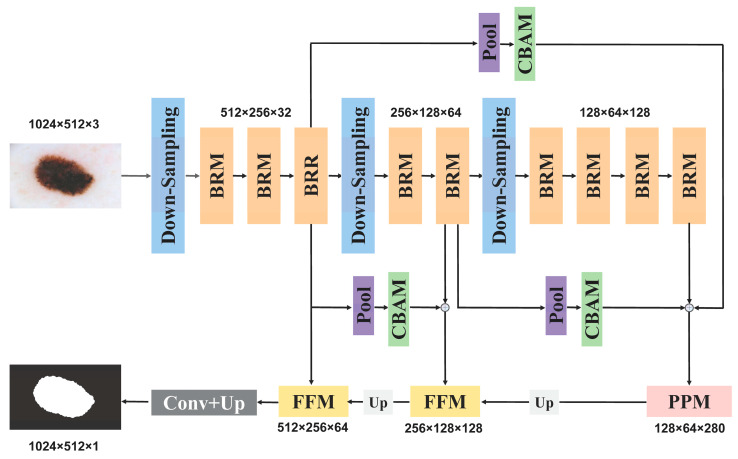
The overall architecture of ELANet.

The network’s overall structure is depicted in Figure 2, and it is divided into three primary layers. Each of these layers conducts feature processing and aggregation through a sequence of modules. The processing details for each layer are elaborated upon below:

**First Level:** At this level, the input image undergoes processing through a downsampling module, reducing its size by half and increasing the channel count to 32. This results in the formation of a feature map with dimensions of 512 × 256 × 32. Then, after three bilateral residual modules, the integration of features is achieved. During the integration process, the size of the feature map and the number of channels are kept constant.

**Second Level:** The feature map is further downsampled to half its previous size, simultaneously doubling the number of channels. This results in the creation of a feature map with dimensions of 256 × 128 × 64.

**Third Layer:** The third layer features a dimension of 128 × 64 × 128 for its feature map. Each of these three differently sized feature maps contains ample global contextual information. To maximize the utility of this information, we employ attention mechanisms to aggregate it, ensuring their effective guidance of feature information during the decoding process.

Finally, aggregate the outputs from the three levels and feed them into the pyramid pooling module to further expand the sensory field. While restoring the feature map size, the feature aggregation module is used, which can merge high-level semantic and spatial information together. Finally, after specific processing, the network outputs the prediction results.

#### 3.1.1. Downsampling Module

**Figure 3:**
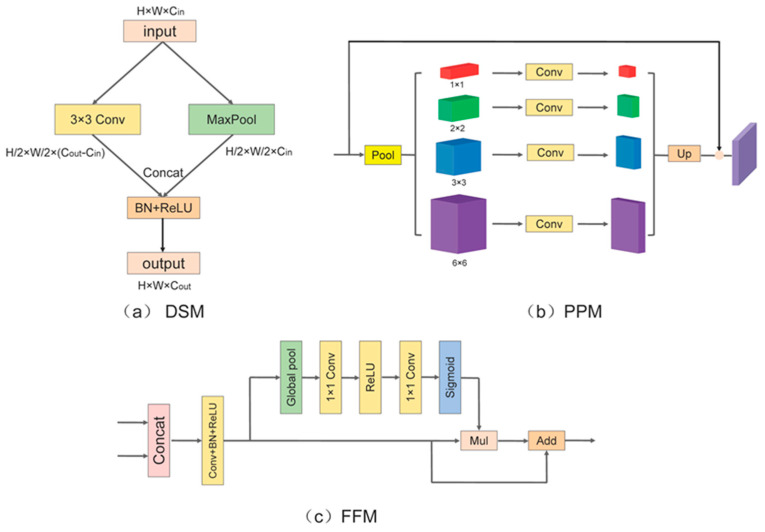
Three modules used in ELANet. (**a**) downsampling module (DSM), (**b**) pyramid pooling module (PPM), (**c**) feature fusion module (FFM).

The downsampling module plays a crucial role in resizing the input graph while augmenting its channel count. This process aims to enhance feature representation and decrease computational complexity, as shown in Figure 3a. For the input feature graph $F\in R^{C_{in}\times H\times W}$, is split into two branches. One branch employs maximum pooling to downsample the size to half, resulting in $F’\in R^{C_{in}\times H/2\times W/2}$. The other branch employs 3 × 3 convolution with a stride of two while controlling the number of output channels as $\left(C_{out}-C_{in}\right)$, producing the output $F’’\in R^{(C_{out}-C_{in})\times H/2\times W/2}$ (where $C_{in}$ and $C_{out}$ represent the number of input and output channels, H and W represent the width and height of the feature map). Afterward, they are merged to get the output using the activation function. Compared with directly using the pooling layer to perform the downsampling operation, this can supplement the feature information and does not add too much computational burden. The specific formula is as follows:

(1) $$DSM\left(F\right)=\rho \left(Concat\left(C_{3\times 3}\left(F\right),MaxP\left(F\right)\right)\right),$$

where $DSM\left(F\right)$ denotes the output of the feature map F after the downsampling module, ρ stands for the BN+ReLU operation, BN is the batch normalization, and ReLU is the nonlinear activation function, while $C_{3\times 3}$ denotes the 3 × 3 convolution, and MaxP is the maximum pooling.

#### 3.1.2. Pyramid Pooling Module

The PPM serves as the core component of PSPNet [11]. It operates by decomposing the input information into four feature maps with sizes of 1 × 1, 2 × 2, 3 × 3, and 6 × 6 following an adaptive pooling operation. Subsequently, they are respectively adjusted to the original 1/4 by a single 1 × 1 convolution, after which they are up-sampled to the initial sizes by bilinear interpolation, summed up, and finally summed up with the residuals to obtain the final aggregated features. The PPM is able to acquire the feature information of different scales and regions and fuse them together, which helps to enhance the model’s understanding of the global contextual information, thus improving the performance of image segmentation, as shown in Figure 3b.

#### 3.1.3. Feature Fusion Module

Feature fusion is a common processing technique, but to enhance the depth of interaction between the two input features, we employ a more comprehensive and effective feature aggregation method [42]. This approach leverages global information to guide the fused module in suppressing irrelevant feature details while improving its capacity to effectively utilize information compared with traditional methods. In Figure 3c, it combines the two given inputs and adjusts the channel count to the desired number using a 1 × 1 convolution. Subsequently, global pooling is applied to incorporate global information, enhancing the network’s sensitivity to features. Following the concept of squeeze and excitation [39], this result is then dot-multiplied with the inputs to recalibrate the response information. Finally, it is summed with the initial signal to produce the aggregated output.

### 3.2. Multiscale Attention Fusion

Multi-scale fusion is increasingly prevalent in detection and segmentation tasks. For lightweight networks with fewer layers, effectively utilizing feature maps at various scales is crucial for improving segmentation performance. However, simple summation might not facilitate efficient information interaction. To convey deep features optimally, we incorporate the lightweight attention mechanism CBAM [40] into the fusion process. This modification allows us to emphasize or suppress specific feature information without introducing additional computational overhead. The module comprises spatial attention and channel attention mechanisms, as outlined below:

**Channel Attention Module:** This module is designed to guide the network’s attention to specific channel-wise feature information. It takes the input feature map and performs average pooling and maximum pooling operations to reduce the feature map’s spatial dimensions. The maximum pooling operation identifies the most significant features in each channel, while the average pooling operation computes the average feature value for each channel. These compressed feature maps are then passed through a multilayer perceptron (MLP) for further processing, allowing the network to learn complex relationships among input features. Finally, the outputs from these operations are fused and passed through an activation function. This module amplifies the model’s response to task-critical features, as described below:

(2) $$CA\left(F\right)=\sigma \left(\mathrm{MLP}\left(AvgP\left(F\right)\right)+\mathrm{MLP}\left(MaxP\left(F\right)\right)\right)$$

where $CA\left(F\right)$ denotes the output of input F through the channel attention module, AvgP and MaxP stand for average and maximum pooling, respectively, and σ denotes the sigmoid function.

**Spatial Attention Module:** This module is designed to assist the model in adapting to variations in feature importance across different spatial locations. Unlike the channel attention module, it sequentially performs average pooling and maximum pooling operations along the channel axis. Subsequently, the module applies a single 7 × 7 convolutional layer, which is capable of learning to identify feature distinctions in various spatial locations, generating attention feature maps. Finally, the results are obtained through an activation function. This module enhances the network’s emphasis on critical regions within the image, thereby enhancing the model’s performance and robustness. The formula is as follows:

(3) $$SA\left(F\right)=\sigma \left(C_{7\times 7}\left(\left[AvgP\left(F\right);MaxP\left(F\right)\right]\right)\right),$$

where $SA\left(F\right)$ denotes the output of input F after the spatial attention module, and $C_{7\times 7}$ denotes the 7 × 7 convolution.

In this paper, the CBAM used in the multiscale attention aggregation section computes the attention map sequentially along both channel and spatial dimensions, which to some extent solves the problem of balancing the model between perceptual details and global context. The specific formula is as follows:

(4) $$\begin{array}{c} F^{\prime }=CA\left(F\right)⊗F,\\ F^{\prime \prime }=SA\left(F^{\prime }\right)⊗F^{\prime }, \end{array}$$

where ⊗ denotes element-by-element multiplication, CA and SA denote channel and spatial attention, respectively, F is the initial input feature map, $F^{\prime }$ is the output feature map of the first step after channel attention, and $F^{\prime \prime }$ is the final output result after CBAM.

### 3.3. Bilateral Residual Module

**Figure 4:**
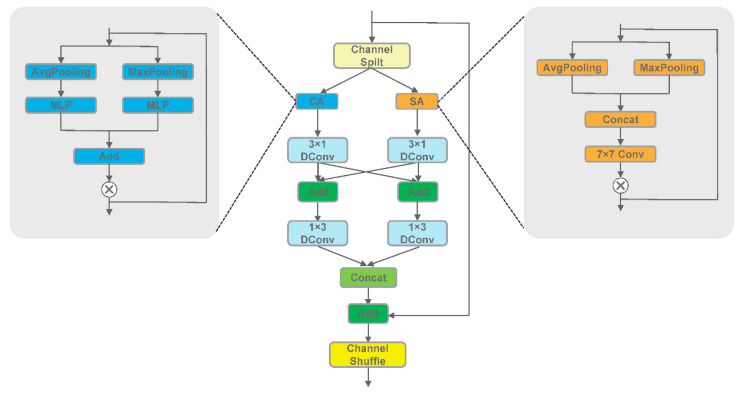
Bilateral Residual Module (BRM).

Lightweight networks must deliver efficient performance with a limited number of layers; an excessively deep backbone structure is not suitable. Therefore, the key to building such a model lies in designing efficient and lightweight components. The split-shuffle-non-bottleneck (SS-nbt) [43] lacks sufficient bilateral information interactions and has limitations in capturing information from various locations in the image. On the other hand, the efficient asymmetric residual (EAR) module [44] and bottleneck residual unit (BRU) [45] incorporate channel attention mechanisms and convolutional blocks at multiple locations, resulting in a more complex overall structure. Additionally, the repetitive use of a single attention mechanism introduces redundancy as the network deepens, potentially affecting feature selection and concentration. To address these concerns, we propose the bilateral residual module (Figure 4) to capture image features more effectively.

Specifically, when an input feature map $F_{in}\in R^{C\times H\times W}$ is passed into the bilateral residual module, it undergoes an initial channel separation operation. This operation, designed to be more computationally efficient and memory-saving compared with standard convolution-based channel separation, is expressed as follows:

(5) $$x_{1},x_{2}=SP\left(F_{in}\right),$$

where $x_{1}$, and $x_{2}$ represent the two results output after channel separation, and SP represents the channel split operation.

In the next process, each of the two branches will pass through the attention mechanism once. It should be noted that the selection of the attention module is not fixed, but two different attention mechanisms are assigned to the left and right branches, i.e., channel attention and spatial attention. The channel attention mechanism enhances fine-grained feature selection by effectively capturing the correlations and importance between different channels within the image. Meanwhile, the spatial attention mechanism aids the network in concentrating on information from various locations in the image, allowing it to better capture spatial, structural, and contextual information. This combination of dual attention mechanisms empowers the model to gain a more comprehensive and accurate understanding of the image content, avoiding the redundant utilization of a single module at different levels. Such a design strategy can focus on the important features in the image more accurately, thus improving segmentation performance. The specific formula is as follows:

(6) $$y_{att1}=CA\left(x_{1}\right),$$

(7) $$y_{att2}=SA\left(x_{2}\right),$$

where $y_{att1}$ and $y_{att2}$ represent the outputs of the left and right branches after the attention mechanism, CA represents the channel attention mechanism, and SA represents the spatial attention mechanism.

Then the sensory field is expanded with null convolution to obtain feature information at long distances. To achieve a more powerful feature extraction capability while maintaining the number of model parameters, an asymmetric convolution strategy is used as an alternative to the traditional 3 × 3 convolution. However, since two branches used in different network layers may suffer from insufficient information sharing, an information-sharing operation is performed between the decomposed convolutional layers to achieve complementarity of features from different branches. This approach is effective in inducing features from different branches to merge to some extent, thus achieving a more integrated feature representation. After the previous steps, we combine the results of the two branches, which are residually summed with the original input. Finally, a channel shuffle operation is performed to obtain the output of the bilateral residual module. The specific formula for the above operation is as follows:

(8) $$y_{1}=DC_{1\times 3,l}\left(DC_{3\times 1,l}\left(y_{att1}\right)+DC_{3\times 1,r}\left(y_{att2}\right)\right),$$

(9) $$y_{2}=DC_{1\times 3,r}\left(DC_{3\times 1,r}\left(y_{att2}\right)+DC_{3\times 1,l}\left(y_{att1}\right)\right),$$

(10) $$F_{out}=SH\left(Concat\left(y_{1},y_{2}\right)+F_{in}\right),$$

where $y_{1}$, $y_{2}$ represent the outputs of the two branches, $DC_{m\times n,l}$ and $DC_{m\times n,r}$ refer to the m×n atrous convolution in the left and right branches, respectively. $F_{out}$ represents the final output feature maps of the bilateral residual module, SH stands for the channel shuffle operation, and Concat is the stagewise concatenation operation.

## 4. Experiment

### 4.1. Dataset

We select the public skin lesion datasets ISIC2016 [46], ISIC2017 [47], and ISIC2018 [48,49] published by the International Skin Imaging Collaboration (ISIC) to evaluate the model, and the thyroid nodule segmentation dataset TN3K for evaluating the model generalization capability. The specific details of each dataset are shown in Table 1.

### 4.2. Evaluation Metrics

We choose two common metrics to assess the model’s performance: mean intersection-over-union (mIoU) and accuracy (Acc). mIoU quantifies the similarity between the predicted results and the ground truth, while Acc is computed as the ratio of correctly predicted samples to the total predicted samples. The formulas for these metrics are as follows:

(11) $$\mathrm{mIoU}=\frac{1}{k+1}\overset{k}{\underset{i=0}{\sum }}\frac{TP}{TP+FP+FN},$$

(12) $$\mathrm{Acc}=\frac{TP+TN}{TP+TN+FP+FN},$$

where *TP* stands for the number of true positives, *FP* stands for the number of false positives, *FN* stands for the number of false negatives, and *TN* stands for the number of true negatives, all of which are parameters in the confusion matrix. *k* is the number of the foreground.

### 4.3. Implementation and Configuration

In the experimental process, we employ the BCE (Binary Cross-Entropy) loss [50] function for training. The optimization is performed using stochastic gradient descent (SGD) as the optimizer. We initialize the learning rate at 0.01 with a momentum parameter of 0.9. To mitigate overfitting and enhance generalization, a weight decay strategy with a decay coefficient of 0.0001 is employed. Additionally, a cosine annealing technique for learning rate reduction is utilized to guide the training process toward optimal convergence. The training epoch is set equal to 1000, and the batch size is 8. 

To further improve the model’s generalization performance and reduce the risk of overfitting, we introduce various data augmentation techniques, including random flipping, Gaussian blurring, and image rotation. Moreover, to maintain a consistent image size and prevent distortion caused by augmentation operations, images are padded with a gray border and resized to a uniform input size of 1024 × 512 pixels. It is worth noting that all experiments are conducted within the PyTorch framework and are trained using an NVIDIA GeForce RTX 3090 Ti graphics card with 24 GB of memory (NVIDIA, Santa Clara, CA, USA). 

### 4.4. Comparisons with State-of-the-Art Methods

To verify the performance of ELANet, we conducted several comparative experiments on the ISIC2016, ISIC2017, and ISIC2018 datasets, respectively, and the specific experimental data are displayed in Table 2. The comparison experiments include some representative medical segmentation algorithms, such as the most classical UNet [13], which is characterized by a compact and symmetric encoder-decoder structure and guarantees the transfer of feature information through jump connections; TransUNet [51] and SwinUNet [52], which combine the ideas of Transformer and CNN in order to better understand the medical image’s semantic information; and the lightweight medical segmentation models MALUNet [35] and EGEUNet [36], which improve the accuracy of the models in understanding and analyzing images by combining the attention mechanism as well as the grouping idea.

**Figure 5:**
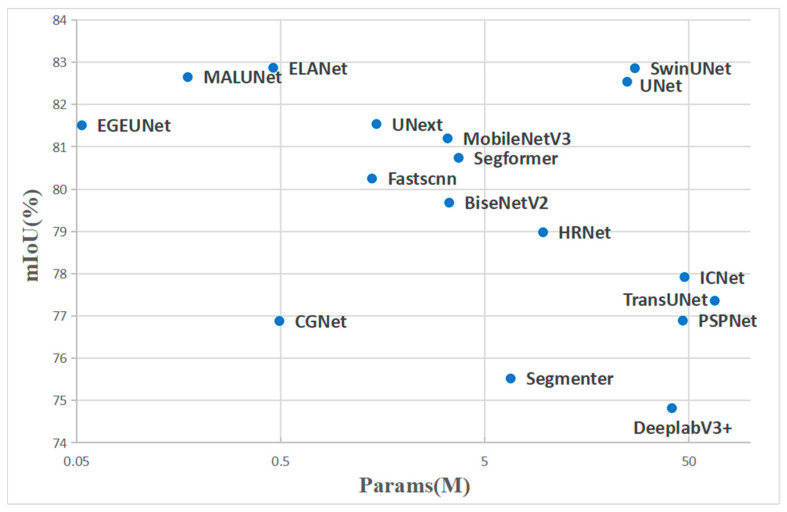
Accuracy-Params comparisons on the skin lesion datasets ISIC2018.

Although MobileNetV3 and UNext have the highest accuracy on the ISIC2016 and ISIC2017 datasets, respectively, when combining the three datasets, they are less accurate on the other datasets, and the models are not stable enough, whereas ELANet excels on all three datasets, with a better average accuracy than the other models. In addition, our model parameter is 0.45 M, which puts very little storage pressure on the device in real deployment. Through the visual analysis in Figure 5, we find that most models have higher parameter counts than ELANet, and the accuracy of models with fewer parameters is lower than that of ELANet; thus, our approach strikes an excellent balance between accuracy and lightweightness.

**Figure 6:**
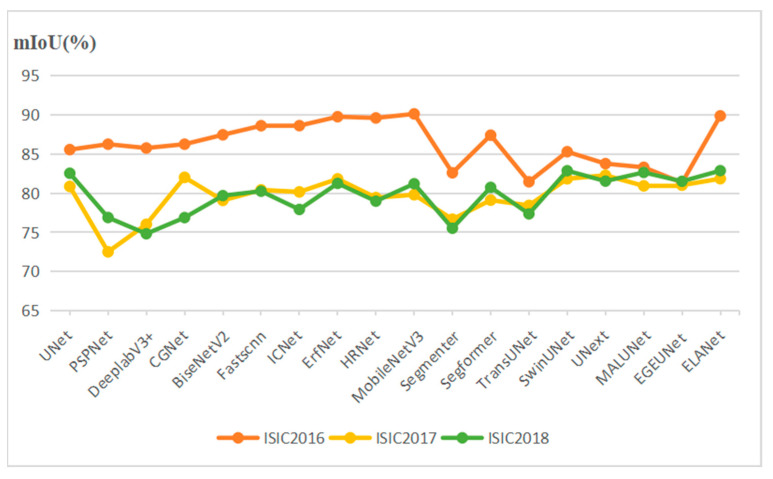
The line charts show the segmentation performance of the different models on the three skin lesion datasets.

In Figure 6, the final results of the above segmentation models on the three datasets are visualized. Looking to see that the models’ prediction accuracy for ISIC2016 is generally higher than that of ISIC2017 and ISIC2018, we speculate that it may be because there is less training data in the ISIC2016 dataset, which leads to easier fitting of the models. Whereas in ISIC2017 and ISIC2018, the accuracy of each model is closer, it may be that when the data features are increased to a certain level, the prediction performance of the model also tends to stabilize.

**Figure 7:**
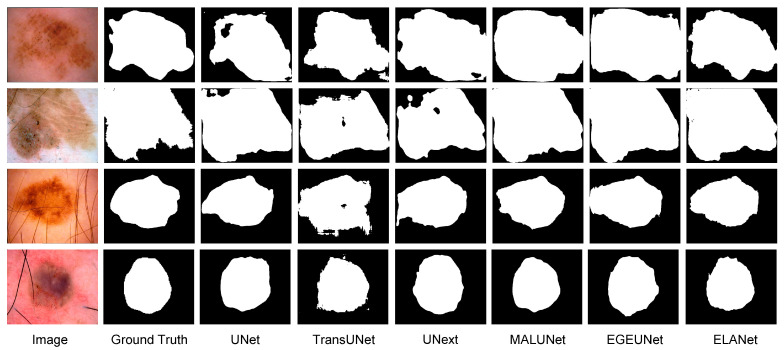
Visual comparisons with different state-of-the-art methods on ISIC2018.

In addition to the numerical comparisons, we show in Figure 7 a visualization image of the prediction results for each model on the ISIC2018 dataset. The first two columns display the input images and their corresponding ground truth labels, while the subsequent columns showcase the predicted images from different models. These visualizations highlight ELANet’s exceptional segmentation capabilities, particularly for skin lesion images with oversized lesion boundaries, blurred boundaries, and irregular shapes. It is clear that ELANet not only identifies more lesion areas compared with other models but also excels at handling edge details. These results further demonstrate the effectiveness of ELANet in the task of skin lesion segmentation, and its superior performance relies on the interplay of the various modules in the ELANet structure, which helps the model to cope with various complex tasks.

### 4.5. Ablation Experiments

To verify that the modules used in the model are valid, we conduct ablation experiments on the ISIC2016 dataset, which are divided into three parts: choice of attentional mechanism in the bilateral residual module, multi-scale attention fusion, and pyramid pooling module. The experimental environments and dataset distributions used by these networks are identical to ensure fairness in the experiments.

**Attention Mechanisms:** Three ways of equipping all spatial attention, all channel attention, and one attention mechanism each for left and right in both branches of the BRM. As seen in Model 1-Model 3 of Table 3, the segmentation accuracy of only configuring the spatial attention mechanism is the lowest, followed by the way of only equipping the channel attention, and the highest is the method of the hybrid attention mechanism, with the mIoU reaching 89.18%, which is improved by 3.55% compared with the lowest way. Importantly, this enhanced performance is achieved with minimal impact on the number of model parameters and computational requirements, highlighting the reasonability and effectiveness of this configuration.

**Multiscale Attention Fusion:** After determining the choice of hybrid attention, we add a multiscale attention fusion method to the model. As shown in Model 4 in Table 3, the segmentation performance of the model has a small improvement, and the number of parameters is only 0.018 M more, which is an acceptable range.

**Pyramid Pooling Module:** As shown in Model 5 in Table 3, after we introduce the PPM. The segmentation precision and accuracy are improved, while the number of parameters and the computation volume are only increased by 0.058 M and 0.442 G.

Finally, in our study, the introduction of MAF and PPM, combined with the hybrid attention mechanism (Model 6 shown in Table 3), led to a notable improvement in segmentation accuracy, achieving a mIoU of 89.87% and an accuracy of 96.78%. Also, this improvement is accomplished without a substantial increase in the number of model parameters or computational requirements, thus meeting the accuracy and lightness criteria for the model. This underscores the effectiveness of selecting the hybrid attention mechanism, which allows for the acquisition of more feature information without significantly adding to the computational burden. Additionally, the introduction of the MAF and PPM, while resulting in a modest increase in parameters and computation, brought substantial performance enhancements to the model. These modules have proven to be effective and viable additions, significantly enhancing the skin lesion segmentation task.

**Figure 8:**
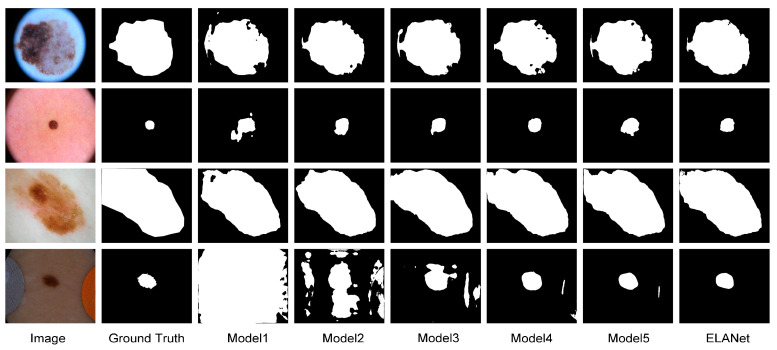
Visualisation comparing ablation experiments in the ISIC2016 dataset.

In Figure 8, visualizations of each model in the ablation experiments are presented. It is evident that in Model 1, when the spatial attention mechanism is selected for the bilateral residual module, the segmentation performance is poor, especially in recognizing target irregularities and in darker environments. In Model 2, after switching to the channel attention mechanism, the prediction ability is slightly improved, but it still struggles with challenging tasks in average lighting conditions.

Model 3, equipped with the hybrid attention mechanism where both types of attention mechanisms work together, enhances the model’s ability to locate the spatial position of the target compared with the method using only one attention module, but there’s still room for improvement in capturing detailed information. By further adding the MAF and PPM in Models 4 and 5, respectively, the model’s ability to process edge detail information is expanded, and it is observed that both methods have a slight improvement in performance.

The final Model 6 (ELANet), which adds both MAF and PPM, exhibits excellent segmentation performance, even for irregular images with blurred edges, and recognition in dark environments. This indicates that our approach effectively addresses these complexities and achieves outstanding performance.

### 4.6. Generalization Experiment

To assess the generalization ability of the proposed method, we finally performed generalization experiments on the TN3K (thyroid nodule segmentation dataset), the results of which are shown in Table 4. From the experimental data, we can see that ELANet does not achieve the highest accuracy. We analyze the reason for this, which is due to the fact that the skin lesion dataset and the thyroid nodule dataset have different data features and distributions, and there are large morphological differences between the lesion regions of the images, thus leading to differences in the performance of the models on the different datasets. But it performs better compared with most models. This proves that ELANet has excellent generalization ability when dealing with different types of medical image tasks, showing its good potential and stability in the field of medical imaging.

## 5. Conclusions

In this paper, we propose an efficient, lightweight attention network for skin lesion segmentation called ELANet. The BRM used in the network can fully extract the image features, and by embedding two different attentional mechanisms into the BRM, two different kinds of detailed information can be generated to complement each other in order to enhance the segmentation performance of the model. Additionally, ELANet employs a MAF strategy to fuse features of various scales, resulting in a global contextual understanding. The use of asymmetric and atrous convolutions helps to reduce parameters while expanding the receptive field, making ELANet suitable for clinical environments with limited computational resources. Finally, the comparison experiments on three public skin lesion datasets show that ELANet can achieve the most excellent segmentation performance at very low parameter counts, outperforming other common segmentation models. ELANet also demonstrates competence in handling complex segmentation tasks, such as those involving irregular regions, blurred boundaries, and oversized boundaries, with great potential to be a good aid to healthcare professionals for accurate diagnosis. 

In the future, optimizing the structure of ELANet and improving the generalization ability of the model so that it can be applied to other medical image processing fields are the main challenges.

## Figures and Tables

**Table 1:** Detailed description of the three skin lesion datasets ISIC2016, ISIC2017, and ISIC2018, and the thyroid nodule dataset TN3K.

Dataset	Training	Validation	Testing
ISIC2016	900	/	379
ISIC2017	2000	150	600
ISIC2018	2594	100	1000
TN3K	2879	/	614

**Table 2:** Comparison with the state-of-the-art methods on three skin lesion datasets.

Method	ISIC2016		ISIC2017		ISIC2018		Params (M)	FLOPs (G)
	mIoU (%)	Acc (%)	mIoU (%)	Acc (%)	mIoU (%)	Acc (%)		
UNet [13]	85.56	95.03	80.86	91.40	82.54	91.95	24.891	225
PSPNet [11]	86.25	95.62	72.51	87.85	76.89	89.41	46.602	357
DeeplabV3+ [10]	85.76	95.52	76.02	89.48	74.82	88.20	41.216	353
CGNet [53]	86.25	95.30	82.02	91.86	76.88	89.17	0.492	6.896
BiseNetV2 [54]	87.45	96.08	79.08	90.69	79.68	90.84	3.341	24.571
Fastscnn [55]	88.61	96.36	80.41	91.32	80.25	90.94	1.398	1.853
ICNet [56]	88.62	96.27	80.16	91.27	77.92	89.52	47.528	30.745
ErfNet [33]	89.76	96.71	81.82	91.96	81.25	91.42	2.082	29.062
HRNet [57]	89.60	96.69	79.43	90.94	78.98	90.45	9.636	37.157
MobileNetV3 [58]	**90.12**	**96.86**	79.80	91.96	81.20	91.55	3.282	8.687
Segmenter [59]	82.60	94.00	76.67	89.32	75.52	87.80	6.685	4.722
Segformer [60]	87.40	95.80	79.11	90.79	80.74	91.05	3.716	3.682
TransUNet [51]	81.45	93.57	78.43	90.23	77.36	89.65	66.815	32.63
SwinUNet [52]	85.29	94.95	81.84	91.88	82.86	**92.21**	27.145	5.91
UNext [34]	83.77	94.44	**82.28**	**92.15**	81.54	91.45	1.471	0.439
MALUNet [35]	83.30	94.11	80.93	91.49	82.65	91.96	0.175	0.083
EGEUNet [36]	81.33	93.21	81.02	91.46	81.51	91.55	**0.053**	**0.072**
ELANet	89.87	96.78	81.85	91.99	**82.87**	92.19	0.459	8.430

Note: bold indicates best performance.

**Table 3:** Ablation experiments on the ISIC2016 dataset.

Method	MAF	PPM	Attention			mIoU (%)	Acc (%)	Params (M)	FLOPs (G)	Time (S/Image)
			SA	CA	HA					
Model 1	×	×	√	×	×	85.63	95.10	**0.270**	6.754	0.0331
Model 2	×	×	×	√	×	87.84	96.06	0.273	**6.679**	**0.0328**
Model 3	×	×	×	×	√	89.18	96.51	0.271	6.716	0.0337
Model 4	√	×	×	×	√	89.24	96.56	0.289	7.260	0.0362
Model 5	×	√	×	×	√	89.29	96.56	0.329	7.158	0.0354
Model 6 (ELANet)	√	√	×	×	√	**89.87**	**96.78**	0.459	8.430	0.0417

Note: MAF: Multi-scale Attention Fusion, PPM: Pyramid Pooling Module, Attention: Attention Mechanisms for Bilateral Residual Modules, SA: Spatial Attention, CA: Channel Attention, HA: Hybrid Attention, M: Million, G: Giga-Billion, bold indicates best performance.

**Table 4:** Generalization experiments on the thyroid nodule dataset.

Method	mIoU (%)	Acc (%)	Params (M)	FLOPs (G)
UNet [13]	84.94	96.21	24.891	225
CGNet [53]	83.17	93.63	0.492	6.896
MobileNetV3 [58]	82.87	95.31	3.282	8.687
Segformer [60]	79.10	84.79	3.716	3.682
TransUNet [51]	**86.86**	96.47	66.815	32.63
SwinUNet [52]	80.57	94.96	27.145	5.91
UNext [34]	86.64	**96.63**	1.471	0.439
MALUNet [35]	82.99	95.65	0.175	0.083
EGEUNet [36]	83.14	95.75	**0.053**	**0.072**
ELANet	85.42	96.30	0.459	8.430

Note: bold indicates best performance.

## Data Availability

The raw data supporting the conclusions of this article will be made available by the authors on request.
